# Correlates of use of withdrawal for contraception among women in Vietnam

**DOI:** 10.1186/s12905-020-00957-z

**Published:** 2020-04-29

**Authors:** Nghia Nguyen, Linh Nguyen, Hoai Nguyen, Maria F. Gallo

**Affiliations:** 1 Department of Obstetrics and Gynecology, Vinmec International Hospital, 458 Minh Khai, Hanoi, Vietnam; 2 Division of Epidemiology, College of Public Health, The Ohio State University, Cunz Hall, 1841 Neil Avenue, Columbus, OH 433210 USA

**Keywords:** Contraception, Vietnam, Pregnancy, unintended, Withdrawal, Women

## Abstract

**Background:**

Despite its relatively low effectiveness, withdrawal is a common contraceptive practice. In Vietnam, health concerns about hormonal contraception are strong and account for substantial method discontinuation. Given the paucity of evidence on withdrawal, our objective was to identify correlates of using withdrawal among women not desiring pregnancy.

**Methods:**

We conducted a secondary analysis of data from a cross-sectional study of sexually-active adult women attending a public hospital in Hanoi, who did not desire pregnancy. We enrolled a stratified sample of women using the intrauterine device, combination oral contraception, or neither method. Participants completed a questionnaire on demographics and reproductive history and behaviors. We used multinomial logistic regression to evaluate correlates of using a tier 3 contraception method (without withdrawal) and using withdrawal (alone or with a tier 3 method) compared to the referent category of using a tier 1 or 2 method (without withdrawal).

**Results:**

Of the 489 participants in the analysis, 52.3% reported using tier 1 or 2 method (without withdrawal); 19.8% reported tier 3 contraception (without withdrawal) and 27.9% reported using withdrawal (alone or with a tier 3 method). Compared to those using a tier 1 or 2 method, women using withdrawal had lower odds of reporting that avoiding pregnancy was very important or important to them (aOR, 0.4; 95% CI, 0.3–0.7). Women using withdrawal had higher odds of reporting that their husband/partner refuses to give them money for household expenses, even when he has the money (aOR, 2.8; 95% CI, 1.4–5.6).

**Conclusions:**

Women using withdrawal might have less relationship power than nonusers. They also might rely on the practice because they are more ambivalent about pregnancy.

## Background

The use of withdrawal (coitus interruptus) predates the introduction of modern methods of contraception and remains today a commonly used method for preventing pregnancy [1, 2]. Worldwide in 2015, withdrawal was the most effective of any contraceptive method used by an estimated 3.1% of reproductive-age women who were married or otherwise in-union [2]. Prevalence varied widely by region, with less than 1.0% reporting relying on withdrawal in Southern Africa, Eastern Asia, Western Europe, and Eastern Africa (0.4, 0.6, 0.6 and 0.8%, respectively). In contrast, 12.9% of women in Southern Europe and 15.1% in Western Asia reported withdrawal to be the most effective contraceptive method that they currently used.

Prevalence figures for withdrawal, though, are sensitive to methods of posing the question and calculating the outcome. First, when asked to report on their contraceptive method use, survey respondents might fail to consider withdrawal as a method unless they are prompted to do so [3–5]. Furthermore, social desirability bias might prevent their reporting reliance on a traditional method to health care providers or researchers [1]. Also, while some use withdrawal alone, withdrawal is often combined with other method(s). For example, couples might use withdrawal routinely with male condoms or periodic abstinence to improve the overall protection for the act [6], or they might use withdrawal to compensate for inconsistent adherence to another method. Some report using withdrawal in place of condoms with established partners whom they perceive to be at low risk of HIV or other sexually transmitted infections (STIs) [5, 7] while others might use withdrawal as a result of misconceptions about the method’s ability to protect against HIV/STI acquisition [8]. Despite the common practice of mixing methods, prevalence estimates often are calculated for the single most effective method used by the participant. These measures, therefore, will not account for many withdrawal users.

Withdrawal is less effective than many other contraceptive methods, with a one-year, probability of pregnancy during typical use of 20% [9]. In comparison, the one-year typical failure rate for oral contraception is 7%. The estimated effectiveness of withdrawal, though, is based on data from the U.S., and the frequency of failure could be expected to differ between settings, in part because the way that withdrawal is practiced could vary substantially between different cultures [10]. Even if performed perfectly at every act, withdrawal theoretically carries a risk of pregnancy because pre-ejaculatory fluid contains sperm [11].

Despite its lower effectiveness compared to modern methods of contraception, withdrawal offers a number of advantages, including its lack of cost and other issues of access. Concerns about side effects and health risks from modern methods of contraception constitute a major barrier to contraception use [12], and some report preferring withdrawal for its lack of health effects, greater intimacy compared to condoms, and “naturalness.” The “naturalness” or non-medical status also is cited as a benefit by those who oppose the use of contraception for religious or cultural beliefs [13]. Finally, although some men describe reduced sexual pleasure from requiring the man to pull out before ejaculation, other men perceive withdrawal as more pleasurable than experiencing the loss in sensation from wearing a condom [14].

Concerns about side effects and health risks from contraception appear to be particularly strong in Southeast Asia, accounting for 36% of non-use among women with unmet need for contraception [12]. These concerns also can lead to method discontinuation; for example, 19% of IUD users in a study conducted in six facilities in Vietnam had discontinued by 24 months, and almost half (49%) of those discontinuing the IUD cited health concerns as the primary reason [15]. Thus, withdrawal might be an acceptable alternative among women in Vietnam; however, evidence on those using withdrawal is sparse. We conducted an exploratory analysis to identify correlates of withdrawal use (either alone or with another relatively low-effective method) among women in Vietnam who did not desire pregnancy.

## Methods

We conducted a secondary analysis of data from a cross-sectional study of 500 adult women, 18 to 45 years of age, who were attending the obstetrics-gynecology department in a large public hospital in Hanoi, Vietnam between November 2017 to September 2018. The study enrolled a stratified, convenience sample of women who currently were using 1) the intrauterine device (IUD); 2) combination oral contraception (COC); or 3) were not using, or seeking to start use of, either method. The primary aim was to evaluate a new approach for measuring women’s beliefs about contraception, and these findings will be reported elsewhere. Participants had to have least a minimal level of literacy and comfort with a computer and be sexually active, which we defined as at least 1 penile-vaginal act in past month. Exclusion criteria consisted of being pregnant, breastfeeding, or desiring pregnancy in the next year. Women provided written consent before enrolling. Institutional review boards at The Ohio State University and the Hanoi School of Public Health approved the study.

Female study interviewers administered a questionnaire in REDCap, which is an electronic data capture tool for collecting and managing study data. The questionnaire covered participant demographics and reproductive, sexual and contraceptive history and related behaviors. The question on current contraception use was as follows: “Are you using any of these methods currently? Pill, IUD, injections, implant, diaphragm, foam, jelly, male partner sterilization, rhythm or periodic abstinence, male condom, female condom, withdrawal, none or other.” All responses were recorded.

Because few women reported using no method (*n* = 9) or reported using withdrawal along with a tier 1 (*n* = 1) or tier 2 method (*n* = 1), we excluded these 11 women from the present analysis. Thus, the analysis was based on 489 participants. We first report the numbers of women who were using each type of contraceptive method stratified by use of withdrawal. We then evaluated potential correlates of contraceptive method use, using three categories: 1) tier 1 or 2 contraceptive method without withdrawal use; 2) tier 3 method without withdrawal use; and 3) any withdrawal (alone or with a tier 3 method). Tier classifications are based on the number of expected pregnancies per 100 women per year using the method with < 1 pregnancy expected for tier 1 methods, 2–7 pregnancies for tier 2 methods, and 18 or greater for tier 3 methods [16].

Given the lack of research on the practice of withdrawal in Southeast Asia, and Vietnam in particular, and given the exploratory nature of this analysis, we evaluated a broad range of potential correlates in the unadjusted analysis. These consisted of the following demographic variables: age, residence (city vs. town or rural area), highest level of education completed (upper secondary or less vs. higher) and monthly household income (< 15,000,000 Vietnamese dong, which was equivalent to about 650 U.S. dollars vs. higher). We also evaluated the following partner and reproductive-related factors history: husband or partner ever refuses to give money for household expenses even if he has the money (yes vs. no or no husband or partner); sexual frequency (at least weekly vs less often); health provider has discussed contraception (yes vs. no); mother or sister has used an IUD or COC (yes vs. no); perception of IUD naturalness (“very natural” or “mostly natural” vs. “mostly unnatural” or “very unnatural”); perception of COC naturalness (“very natural” or “mostly natural” vs. “mostly unnatural” or “very unnatural”); importance of avoiding pregnancy (“very important” or “important” vs. “neutral / no opinion” or “not very important”) and history of forced sex (yes vs. no). Many of these factors (i.e., age, residence, education, income, financial empowerment and sexual coercion) were selected because they were previously identified as correlate of withdrawal use; the remaining were selected for evaluation in this exploratory analysis based on literature suggesting that women use withdrawal because they do not find other methods accessible or acceptable [7, 17–23].

We used multinomial logistic regression to identify correlates of using a tier 3 method (without withdrawal) and using any withdrawal (alone or with tier 3 method) compared to the referent category of using a tier 1or 2 contraceptive method (without withdrawal). All correlates that varied by method use in unadjusted multinomial analysis, based on a *p*-value of ≤0.25, were included in the final adjusted multinomial regression model [24]. We conducted analyses in SAS, version 9.2 (SAS Institute Inc., Cary, North Carolina).

## Results

Participants (*n* = 489) reported using tier 1 or 2 method without withdrawal (52.3%; most often IUD or COC use); tier 3 contraception without withdrawal (19.8%) or withdrawal alone or with a tier 3 method (27.9%) (Table 1). Only 32 women relied on withdrawal alone; most using withdrawal also used another method, most commonly male condoms or rhythm or periodic abstinence. Participants were mostly married (96.5%), were urban (90.4) and of Kinh ethnicity (94.9%). The mean age of participants was 34.0 years (standard deviation, 5.2; range). Age terciles were approximately 21–31, 32–36 and 37–45 years.

**Table 1:** Contraceptive method by withdrawal use among sexually-active women not desiring pregnancy, Hanoi, Vietnam (*N* = 489)

Contraceptive method^a^	No.
Tier 1 or 2 method, no withdrawal	
Implant	5
Intrauterine device	128
Tubal ligation	1
Injection	1
Oral contraception	125
Tier 3, no withdrawal	
Male condom	88
Female condom	5
Foam, jelly, film	0
Rhythm, periodic abstinence	13
Withdrawal with or without tier 3 method	
Male condom	81
Female condom	3
Foam, jelly, film	1
Rhythm, periodic abstinence	70
Withdrawal only	32

^a^Per tier classification by the World Health Organization [15]; women could report multiple contraception methods

In the unadjusted analyses, 5 factors were associated with withdrawal (at the higher *p*-value of 0.25 for this initial assessment): age, husband/partner ever refuses to give money for household expenses even if he has the money, health provider has discussed contraception, importance of avoiding pregnancy, and history of forced sex (Table 2). The adjusted multinomial regression model, which was fitted with these 5 variables, showed 2 factors to be associated with using withdrawal (Table 3) compared to using a tier 1 or 2 method. First, women using withdrawal had higher odds of reporting that their husband or partner refuses to give them money for household expenses, even when he has the money (aOR, 2.8; 95% CI, 1.4–5.6). Second, women using withdrawal also had lower odds of reporting that avoiding pregnancy was very important or important to them (aOR, 0.4; 95% CI, 0.3–0.7).

**Table 2:** Participant demographics, characteristics and behaviors by contraceptive method use^a^ among sexually-active women not desiring pregnancy, Hanoi, Vietnam (*N* = 489)

	Tier 1 or 2 method, no withdrawal (*N* = 257)		Tier 3 method, no withdrawal (*N* = 97)		Withdrawal only or with Tier 3 method (*N* = 135)		*p*-value
	No.	(%)	No.	(%)	No.	(%)	
Age, mean (standard deviation)	34.5	(4.9)	32.9	(5.5)	33.9	(5.6)	0.03
Residence							
City	235	(91.4)	87	(89.7)	120	(88.9)	0.69
Town or rural area	22	(8.6)	10	(10.3)	15	(11.1)	
Highest level of education completed							
Upper secondary or less	64	(24.9)	27	(28.1)	29	(21.5)	0.51
Higher	193	(75.1)	69	(71.9)	106	(78.5)	
Monthly household income							
< 15,000,000 Vietnamese dong	54	(23.7)	15	(17.4)	26	(21.0)	0.48
≥ 15,000,000 Vietnamese dong	174	(76.3)	71	(82.6)	98	(79.0)	
Husband/partner ever refuse to give money for household expenses even if has money							
Yes	18	(7.0)	9	(9.3)	25	(18.5)	< 0.01
No or not applicable	239	(93.0)	88	(90.7)	110	(81.5)	
Sexual frequency							
At least weekly	215	(87.4)	77	(83.7)	108	(82.4)	0.39
Less than weekly	31	(12.6)	15	(16.3)	23	(17.6)	
Health provider has discussed contraception							
Yes	158	(61.7)	54	(56.3)	72	(53.3)	0.25
No	98	(38.3)	42	(43.8)	63	(46.7)	
Mother or sister used the IUD or COC							
Yes	193	(75.4)	67	(69.1)	96	(71.1)	0.42
No	63	(24.6)	30	(30.9)	39	(28.9)	
Perception of IUD naturalness							
Very natural, mostly natural	154	(60.9)	48	(52.2)	80	(60.6)	0.32
Mostly unnatural, very unnatural	99	(39.1)	44	(47.8)	52	(39.4)	
Perception of COC naturalness							
Very natural, mostly natural	155	(60.6)	54	(55.7)	84	(64.1)	0.43
Mostly unnatural, very unnatural	101	(39.5)	43	(44.3)	47	(35.9)	
Importance of avoiding pregnancy							
Very important, important	224	(87.5)	79	(83.2)	104	(77.0)	0.03
Neutral/no opinion, not very important	32	(12.5)	16	(16.8)	31	(23.0)	
History of forced sex							
Yes	22	(8.6)	11	(11.5)	20	(15.0)	0.15
No	234	(91.4)	85	(88.5)	113	(85.0)	

*COC* combination oral contraception, *IUD* intrauterine device

^a^Per tier classification by the World Health Organization [15]

**Table 3:** Correlates of use tier 3 method (no withdrawal) or use of withdrawal (only or with Tier 3 method) compared to use of a tier 1 or 2 method (no withdrawal)^a^ among sexually-active women not desiring pregnancy, Hanoi, Vietnam (*N* = 489)^b^

	Tier 3 method, no withdrawal		Withdrawal only or with Tier 3 method	
	aOR	(95% CI)	aOR	(95% CI)
Age in years	1.0	(0.9, 1.0)	1.0	(1.0, 1.0)
Husband/partner ever refuse to give money for household expenses, even when has money				
Yes	1.4	(0.6, 3.3)	2.8	(1.4, 5.6)
No or not applicable	Ref		Ref	
Health provider has discussed contraception				
Yes	0.8	(0.5, 1.2)	0.7	(0.4, 1.0)
No	Ref		Ref	
Importance of avoiding pregnancy				
Very important, important	0.7	(0.4, 1.1)	0.4	(0.3, 0.7)
Neutral / no opinion, not very important	Ref		Ref	
History of forced sex				
Yes	1.3	(0.6, 3.0)	1.7	(0.8, 3.4)
No	Ref		Ref	

COC = combination oral contraception; IUD = intrauterine device

^a^Per tier classification by the World Health Organization [15]

^b^Results are from full multinomial regression model that includes all variables in the table with Tier 1 or 2 method without withdrawal as the referent

## Discussion

Among the study population of sexually-active women in Hanoi who did not desire pregnancy in the next year, two factors were associated with use of withdrawal: reporting that one’s husband or partner ever refuses to give money for household expenses even if he has the money, and not perceiving avoiding pregnancy in the next year as very important or important. A previous study on the quality of family planning in Vietnam found that use of any withdrawal appeared to be higher among those of Kinh ethnicity versus those of other ethnicities (12.2% versus 8.8%, respectively) [25]. However, we could not evaluate the role of ethnicity because our study was almost exclusively comprised of women of Kinh ethnicity. Age also previously was found to be positively associated with any withdrawal use: 11.6% of women 20–49 years of age reported using any withdrawal compared to 5% of women 15–19 years [25]. In contrast, we did not find evidence suggesting increased use of withdrawal among older women.

Withdrawal appeared to be a common practice in the study population. Among women using withdrawal in the present study, most also reported using either male condoms or a fertility-based awareness method. We did not collect data, though, on whether the multiple methods were combined during single acts, whether women switched between methods for different acts, or the frequency of use or adherence to the use of the methods. Future research could seek to better understand the patterns of withdrawal use within individual women and the reasons for using withdrawal alone versus in combination with other methods.

According to data from the nationally-representative Multiple Indictor Cluster Surveys conducted among reproductive-age, married or cohabiting women in Vietnam, only 5.4% of women in 2014 reported using withdrawal *only* to delay or prevent pregnancy [17]. In a more recent study of family planning services conducted in 2015 in Vietnam, the prevalence of *any* withdrawal use (i.e., alone or combined with other methods) was 11.5% among married women [25]. This figure differed by region, ranging from 7% of women in the Red River Delta region to 17.7% in the North and South Central Coast region.

Future research should seek to address women’s concerns about the safety of highly-effective contraception [12, 15]. At the same time, efforts also should focus on ensuring that couples who opt to use withdrawal receive counseling on the concomitant use of other methods as a harm reduction method for reducing the risk of unintended pregnancy. Although not statistically significant, lower proportions of women using withdrawal in the present study reported that a health provider discussed contraception with them compared to non-users of withdrawal. Withdrawal users should be counseled on the method’s lack of protection against HIV/STI transmission. Because withdrawal users may not perceive withdrawal to be a legitimate method of contraception, they may hesitate to discuss the method during contraceptive counseling with their health care provider [18]. Providers should questions patients specifically about the method rather than make assumptions about its nonuse.

Recent studies have suggested that because most participants combined withdrawal with a hormonal or long-acting method or condoms, withdrawal users might actually represent a subset of individuals who are more proactively protecting themselves against pregnancy compared to nonusers of withdrawal [7, 19]. However, we cannot assess this in the present study as we do not know whether women who reported multiple methods used them as dual protection or whether they switched between two methods on a per act basis. Unlike the earlier U.S. study, few (*n* = 2) women in the parent study used for the present analysis reported using withdrawal and a tier 1 or 2 method. Thus, achieving high contraceptive protection did not appear to be an important motivation for withdrawal use among women in the present study.

Study limitations include limited generalizability as the study sample was a relatively homogeneous group of women – in terms of ethnicity, urbanicity and marital status – attending a single site in Hanoi. Study findings might not apply more broadly to women in Vietnam or to those at highest risk of unintended pregnancy in Vietnam [26]. Furthermore, the cross-sectional nature of this secondary analysis does not allow for establishing a causal relationship between the correlates identified and the practice of withdrawal. Finally, because we selected on contraceptive method status (IUD, COC versus neither) among a convenience sample of women attending a single facility, our results cannot be used to estimate the overall prevalence of withdrawal use among women in Vietnam. Strengths of the analysis include the collection of detailed information on participant characteristics and behaviors that were possibly related to withdrawal in a population that lacks previous research on this topic.

## Conclusion

Study findings indicate that withdrawal might be more common among women with lower relationship power (as evidenced by lack of ability to access funds from their partner). Although the study was restricted to women who reported not desiring pregnancy in the next year, women varied in the degree to which they perceived avoiding pregnancy to be important. Findings suggest that a subset of women might choose to rely on the less-effective method of withdrawal because of their ambivalence about future pregnancy. Future research on withdrawal should determine couple’s motivation for using withdrawal in order to separate out subgroups that likely differ in important ways.

## Data Availability

The study dataset is available from the corresponding author following institutional approvals.

## Abbreviations

aOR: Adjusted odds ratio
CI: Confidence interval
COC: Combination oral contraception
IUD: Intrauterine device
OR: Odds ratio
STI: Sexually transmitted infections
